# Accuracy of Acuros$^{\text {TM}}$ BV as determined from GATE monte-carlo simulation

**DOI:** 10.1007/s13246-022-01190-8

**Published:** 2022-10-27

**Authors:** Tyrone Te Ruruku, Felix Wong, Steven Marsh

**Affiliations:** 1 Medical Physics, Waikato Regional Cancer Center, Hamilton, Waikato New Zealand; 2 Medical Physics, University of Canterbury, Christchurch, Canterbury New Zealand

**Keywords:** Acuros$^{\text {TM}}$ BV, GATE, Brachytherapy

## Abstract

The American Association of Physicists in Medicine’s Task Group No.43 has provided a standardised dose calculation methodology that is now the international benchmark for all brachytherapy dosimetry publications and treatment planning systems. However, limitations in this methodology has seen the development of Model-Based Dose Calculation Algorithms (MBDCA). In 2009, Varian (Varian Medical Systems, Palo Alto, CA, USA) released Acuros$^{\text {TM}}$ BrachyVision (ABV) which calculates dose by explicitly solving the Linear Boltzmann Transport Equation. In this study we have assessed the accuracy of ABV dose calculations within a range of materials relevant to high dose rate brachytherapy with an iridium-192 ($^{\text {192}}$Ir) source. Accuracy assessment has been achieved by implementing a modelled GamaMed Plus $^{\text {192}}$Ir source within a series of phantoms using the GEANT4 Application for Emission Tomography (GATE) to calculate dose for comparison with dose as determined by ABV. Comparisons between GATE and ABV were made using point-to-point profile comparisons and 1D gamma analysis. Source validation results yielded good agreement with published data. Spectrum and TG43U1 comparisons showed no major differences, with TG43U1 comparisons agreeing within ± 1%. Point-to-point comparisons showed large differences between GATE and ABV near the source and in low density materials. 1D gamma analysis pass criteria of 2%/1 mm and 2%/2 mm yielded passing rates ranging between 51.72–100% and 62.07–100% respectively. A critical analysis of this study’s results suggest that ABV is unable to accurately calculate doses in low density materials. Furthermore, spatial accuracy of dose near the source is within 2 mm.

## Introduction

The American Association of Physicists in Medicine’s (AAPM) Task Group 43 published their report on dosimetry of interstitial brachytherapy sources, globally referred to as TG43 [1], in 1995. Following this, a revised dosimetry protocol for brachytherapy dose calculations TG43U1 [2] was published in 2004. TG43U1 provides dosimetry data for additional source data sets, guidelines for experimental and MC determination of reference quality dose distributions, recommendations for acquiring dosimetry data and clinical implementation and has eliminated inconsistencies and omissions in the original TG43 document. The general 2D formalism presented in TG43 was retained in TG43U1, where all seeds and dose distributions surrounding a source are cylindrically symmetrical with the origin of the co-ordinate system at the centre of the active core. TG43U1 provides a standardised dose calculation methodology that is now recognised as a *de facto* international standard [3]. However as discussed thoroughly in the AAPM’s Report of task group 186 (TG186) [4], intrinsic limitations of the TG43 formalism leads to systematic errors in delivered dose. Numerous studies have investigated the accuracy of TG43 and highlighted its shortcomings. Dose differences between $D_{w,w-TG43}$ and MC-calculated $D_{m,m}$ at higher energies (i.e., $^{\text {192}}$Ir) have been shown to range between 2 and 23% when comparing plans for treatments sites such as the esophagus, breast and rectum [5–9].

A possible solution for TG43 is Varian’s (Varian Medical Systems, Palo Alto, CA, USA) Acuros$^{\text {TM}}$ BrachyVision (ABV), a Model-Based Dose-Calculation Algorithm (MBDCA) [10]. Following ABV’s release in 2009, various authors investigated the use of MBDCAs in brachytherapy applications. In 2010, Zourari et al. [11] published the first of a three-paper series which aimed to assess the dosimetric accuracy of ABV. Using the MCNPX general purpose radiation transport MC code, this paper presents comparisons with associated TPS calculated dosimetry for three different $^{\text {192}}$Ir brachytherapy source designs (VS2000 HDR, GMPlus HDR, and GMPlus PDR) in a homogeneous and bounded water phantom. In 2011, Petrokokkinos et al. [12] published the second paper in this series. Using the MCNP5 MC code, this paper presents comparisons with associated TPS calculated dosimetry in a homogeneous water phantom using a GM11004380 applicator with a 90$^{\circ }$ or a 180$^{\circ }$ partial shielding. lastly, in 2013, Zourari et al. [13] published the final paper of the series. Again, using the MCNP5 MC code, the authors present comparisons with associated TPS calculated dosimetry for simulation models prepared from CT DICOM image series of computational models imported to the TPS. Ma et al. [14] also carried out similar work to Petrokokkinos et al. using a variety of MC codes which included ALGEBRA, BrachyDose, egs_brachy, GEANT4, MCNP6 and Penelope2008. All aforementioned studies assume charged-particle equilibrium (CPE) is always present in all voxels. Subsequently they have used track length estimators to score collisional kerma which can only be equated to dose when CPE is present. This is generally a valid assumption in brachytherapy applications; however it is not a valid assumption near the source or at tissue interfaces.

Use of Linear Boltzmann Transport Equation (LBTE) solvers are still in their infancy but are expected to play an important role in future treatment planning system calculations of dose [15]. Clinical implementation of LBTE solvers still require understanding of their implementation and limitations in a clinical setting; therefore the primary aim of this study was to investigate the accuracy of ABV dose calculations in various materials relevant to high dose rate (HDR) brachytherapy using the GEANT4 Application for Emission Tomography (GATE) [16]. Accuracy assessment was achieved by first constructing and validating a MC model of an $^{\text {192}}$Ir GammaMed Plus (GMP) HDR source. Thereafter, the modelled source was used to calculate dose within a series of phantoms using GATE. Comparisons between GATE and ABV output were made using point-to-point profile comparisons and 1D gamma analysis [17]. It shall be noted that the types of tests carried out in this work are considered TG186 mandatory level 2 MBDCA commissioning tests [4]. Concurrently this study also investigated the feasibility of using GATE in brachytherapy applications. GATE is an advanced open source application software developed by the international OpenGate community. Well known for its widespread use in imaging simulations, GATE has also shown promise when used in dosimetry applications [18]. The GATE application encapsulates all complex functionalities of GEANT4 while providing a user friendly interface. It is a multi-layered structure with the base of the structure being the GEANT4 MC code [16];

## Methods and materials

### $^{\text {192}}$r GMP HDR source

**Fig. 1:**
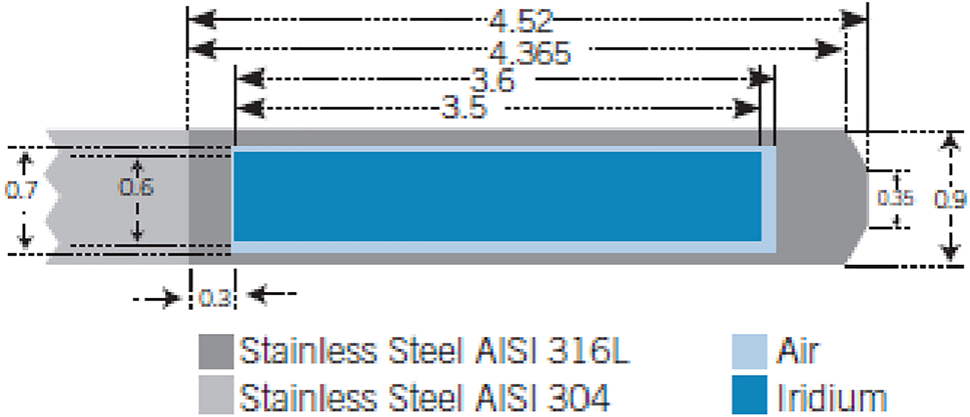
GammaMed Plus source geometry [19]. This image was reproduced with author’s permission

The source used in this study is the GMP source. Figure 1 shows the dimensions, in millimeters, and materials used to model the GMP source [19], with material compositions provided in Table 3. The core of the GMP source is a 3.5 mm long and 0.6 mm diameter solid cylinder of pure iridium. Encapsulating the source core is a hollow AISI 316L stainless steel cylinder, with a density of 8.03 g/cm$^{3}$, an outer diameter of 0.9 mm, an inner diameter of 0.7 mm and a truncated cone end. The source wire is an AISI 304 stainless steel cylinder, with a density of 5.6 g/cm$^{3}$, a diameter of 0.9 mm and a length of 2 mm, which is the maximum length where significant bending of the wire doesn’t occur [10].

Evenly distributed through the pure iridium core is the radionuclide iridium-192 ($^{\text {192}}$Ir). $^{\text {192}}$Ir has a half life of  74 days and decays into platinum-192 via electron capture 95.13$\%$ of the time and $\beta ^{-}$ into osmium-192 the remaining 4.87$\%$. The photon spectrum ranges from 7.82 keV to 1.378 MeV and has an average energy of approximately 370 keV [1, 20]. Simulations for $^{\text {192}}$Ir decay in this study used the $^{\text {192}}$Ir spectrum provided by the Nuclear National Data Center (NNDC) [21].

### GATE setup

The software tool used in this study was the GATE MC toolkit V8.0 built on the GEANT4 v10.4 environment [16]. Both GEANT4 and GATE were built and installed on a PC with an Intel$^\circledR$ Xeon$^\circledR$ E5620 processor. This processing unit is a 4 core processor with a base frequency of 2.4 GHz. Using a single processor resulted in simulation times ranging between 2 and 5 days.

GATE was used to model Varian’s GMP source using geometry and material information acquired from work done by Lopez et al. (Fig. 1) [19]. Following recommendations provided by Rivard et al. [20], the $^{\text {192}}$Ir spectrum attached to the active length of the source was that provided by the National Nuclear Data Center (NNDC) [21].

As the NNDC defined $^{\text {192}}$Ir spectrum has a maximum energy of approximately 1.38 MeV the primary interactions to be considered when modelling the GMP source are Compton scattering and the photoelectric effect. In this study, interactions were modelled in GATE using the *emstandard* Opt0 physics package. For $^{\text {192}}$Ir energies, this package calculates cross sectional data using the *Klein-Nishina* and *Livermore* models for photon interactions, and the *urban* model for electron interactions [22].

GATE V8.0 has three different random number generators (RNG’s) available for use, the *Ranlux64*, the *James Random* and the *Mersenne Twister* (MT).The MT random number generator was used in this study because of its extremely long period of $2^{19937}-1$ [23, 24]. In addition to MT’s extremely long period, the MT generator pre-regenerates a pool of numbers to be used instead of generating random numbers on the fly reducing simulation times [25]. Simulation times were further reduced by using variance reduction techniques (VRT). Following recommendations by Perez et al. [26], a kerma approximator was used and photon energy cut was set to 10 keV.

### Source validation

The GMP source modeled in GATE was validated by comparing simulated photon spectra and TG43U1 factor results with well-validated published data [26–28]. Simulations in this study for all TG43U1 parameters were defined according to recommendations provided by Perez et al. [26], Lopez et al. [19] and Thiam et al. [29]. With the exception of air kerma strength and photon spectrum measurements, where the *EnergySpectrumActor* was used, all TG43U1 dose measurements were acquired using GATE’s kerma approximator, the *TLEDoseActor*. The number of histories for all TG43U1 measurements was set to $2.1 \times 10^{9}$ hist, which is approximately the maximum number of histories available in a single GATE simulation.

### Digital phantom

Dose calculation comparisons were made using a simple digital phantom with interchangeable slabs. This phantom is comprised of four individual slabs of material, where slabs named backscatter 1 (B1) and backscatter 2 (B2) are 101 mm $\times$ 101 mm $\times$ 101 mm and source slab (SS), medium 1 (M1) and medium 2 (M2) slabs are 101 mm $\times$ 101 mm $\times$ 11 mm. With the source centered and positioned parallel to the x-y SS plane, the slabs were combined in various ways to produce six differing phantoms consisting of varying materials. Dose was measured using the standard GATE *DoseActor* returning dose to medium which was then compared with ABV, where ABV also returned dose to medium. The dose calculation grid across the entire system in both GATE and ABV were made up of 1 mm$^{\text {3}}$ voxels. Presented in Table 1 are the materials assigned to each of the various phantom configurations, and material composition data used in both systems is given in Table 3. Monte carlo output doses have been calibrated to mimic clinical brachytherapy doses using a MC calibration factor. The MC calibration factor calculated and applied in this study was determined to be $f = 1.15 \times 10^{12}$ hist/s with an expanded combined uncertainty of *f* is 0.21% (k =2). Doses are presented in this study with geometry factors extracted (i.e., $D \times \frac{G_{0}}{G}$), where geometry factors were calculated using TG43’s line source approximation.

**Table 1:** Phantom material combinations used for GATE vs Acuros BV comparisons

Phantom	BS1	M1	SS	M2	BS2
1	Water	Peek	Water	Air	Water
2	Water	Cartilage	Water	Bone	Water
3	Water	Titanium	Water	Lung	Water
4	Water	Adipose	Water	Muscle	Water
5	Water	Water	Stainless Steel	Water	Water
6	Water	Water	PMMA	Water	–

## Results

### Source validation

Central to our ABV assessment is the simulation of the encapsulated $^{192}$Ir source. The energy weighted photon spectra simulated in this study, and that from Taylor and Rogers [27] in their TG43U1 parameter database is in good agreement. The simulation of the source was therefore considered sufficiently accurate for use in assessing ABV. However, it was observed that although both spectra show the same characteristic X-rays, the fluence of these X-rays is predicted to be less with Taylor and Rogers than from modern versions of GATE which utilize more recent GEANT4 data-sets.

TG43U1 factors simulated in this study, and by Taylor and Rogers [27], Ballester et al. [28] and Perez et al. [26] are also good agreement, with the dose rate constant (DRC), radial dose function and anisotropy data points located between 8 and 172 deg agreeing within 1%.

### GATE determined accuracy of ABV

**Fig. 2:**
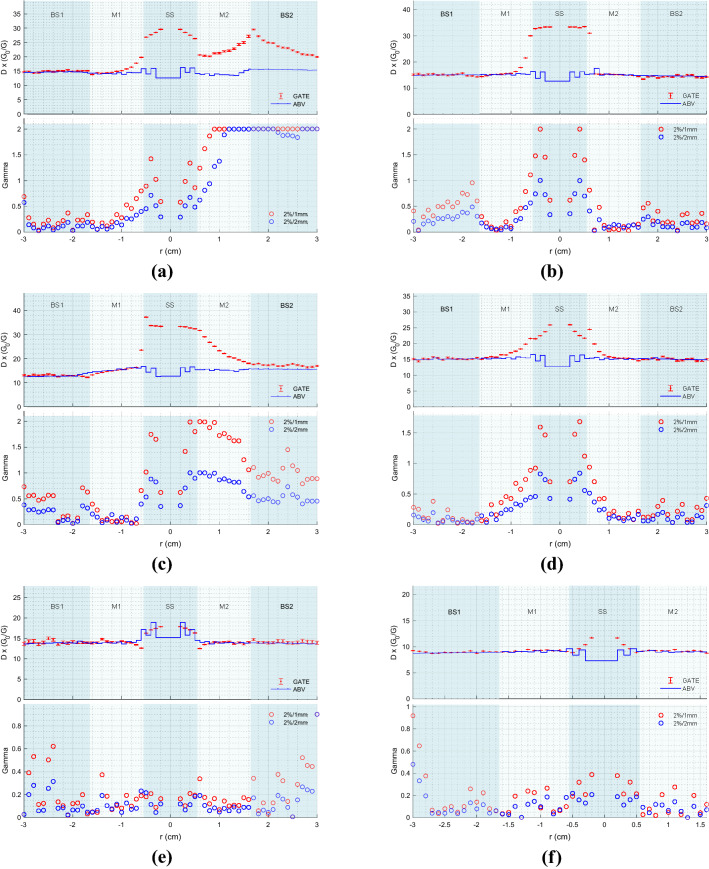
Point-to-point profile comparisons, with associated gamma analyses results using gamma criteria 2%/1mm and 2%/2mm for a source centered in the source slab (SS). **a** Profile comparisons for a phantom made up of water (BS1), peek (M1), water (SS), air (M2) and water (BS2). **b** Profile comparisons for a phantom made up of water (BS1), cartilage (M1), water (SS), bone (M2) and water (BS2). **c** Profile comparisons for a phantom made up of water (BS1), titanium (M1), water (SS), lung (M2) and water (BS2). **d** Profile comparisons for a phantom made up of water (BS1), adipose (M1), water (SS), muscle (M2) and water (BS2). **e** Profile comparisons for a phantom made up of water (BS1), water (M1), stainless steel (SS), water (M2) and water (BS2). **f** Profile comparisons for a phantom made up of water (BS1), water (M1), PMMA (SS) and water (M2)

To assess the accuracy of ABV, dose calculations as calculated by ABV were compared to MC using two comparison techniques. The first of these assessed dose to medium profiles for each of the phantom arrangements as determined by the two dose calculation methods. Plots of these profiles are presented in Fig. 2. The expanded combined uncertainty for all data points is $\le$3.01% (k=1), with larger uncertainties being associated with points positioned further from the source. Dose estimated between −0.2 cm$\le$y$\le$0.2 cm are within or very near to the source, and therefore have no clinical significance. For this reason, comparisons were not made in this region. The second approach to assess accuracy of ABV considered gamma passing rates of a 1-dimensional gamma analysis [17] of values at each point (Fig. 2). Gamma pass rates are tabulated in Table 2. It should be noted that the MatLAB function used in this analysis has a limit parameter that determines how far the function will search when computing gamma. This also therefore specifies the maximum gamma index value. In this study, the maximum obtainable gamma value was set to 2.

**Table 2:** 1D gamma pass rate results for all gamma analyses

Phantom	2%/1mm (%)	2%/2mm (%)
1	51.72	62.07
2	87.93	96.55
3	62.07	94.83
4	91.38	100
5	100	100
6	100	100

**Table 3:** Materials and atomic compositions of the source [19] and digital phantoms [10]

Material	Elemental composition (% mass)	Mass Density (g/cm$^3$)
Titanium	Al(6%), Ti(9%), V(4%)	4.42
Lung	H(10.1278%), C(10.231%), N(2.865%), O(75.7072%), Na(0.184%), Mg(0.073%), P(0.08%), Si(0.225%), Cl(0.266%), K(0.194%), Ca(0.009%), Fe(0.037%), Z(0.001%)	0.26
Muscle	H(10.0637%), C(10.783%), N(2.768%), O(75.4773%), Na(0.075%), Mg(0.019%), P(0.18%), Si(0.241%), Cl(0.079%), K(0.302%), Ca(0.003%), Fe(0.004%), Z(0.005%)	1.05
Bone	H(4.7234%), C(14.433%), N(4.199%), O(44.6096%), Mg(0.22%), P(10.497%), Si(0.315%), K(20.993%), Z(0.01%)	1.92
Adipose	H(11.9477%), C(63.724%), N(0.797%), O(23.2333%), Na(0.05%), Mg(0.002%), P(0.016%), Si(0.073%), Cl(0.119%), K(0.032%), Ca(0.002%), Fe(0.002%), Z(0.002%)	0.92
Cartilage	H(9.6%), C(9.6%), N(2.2%), O(74.4%), Na(0.5%), P(2.2%), Si(0.9%), Cl(0.3%)	1.1
Stainless Steel	C(0.08%), P(0.045%), Cr(19%), Mn(2%), Fe(68.375%), Ni(9.5%)	8
Peek	H(4.1954%), C(79.1557%), O(16.6489%)	1.31
PMMA	H(8.0542%), C(59.984%), O(31.9618%)	1.19
Water	H(11.1894%), O(88.8106%)	1
Air	H(0.0732%), C(0.0123%), N(75.0325%), O(23.6077%), Ar(1.2743%)	0.0012
Vacuum	H(100%)	0.000001
Seed	Ir(100%)	22.42
304	C(0.08%), N(0.1%), Si(0.75%), P(0.045%), S(0.03%), Cr(19%), Mn(2%), Fe(68.745%), Ni(9.25%)	5.6
316L	C(0.03%), N(0.1%), Si(0.75%), P(0.045%), S(0.03%), Cr(17%), Mn(2%), Fe(65.545%), Ni(12%), Mo(2.5%)	8.03
Iridium seed	Ir(100%)	22.42

## Discussion

The primary aim of this study was to assess accuracy of ABV using GATE. In order to have confidence in any findings, significant effort was put in to ensure the performance of GATE and that the simulated source was sufficiently representative of $^{\text {192}}$Ir decay. Once tested the source was included in the various phantoms as described in Sect. Digital Phantom. Results from these analyses will be discussed in terms of clinical relevance.

### Source validation

Crucial to this investigation is the simulation of an accurate GMP source. Validation of this simulation was achieved by comparing simulated energy weighted photon spectra with results produced by Taylor and Rogers [27], and simulated TG43U1 factors with recommended consensus data published by Perez et al. [26]. Comparisons of the energy weighted spectra show there is good agreement between GATE generated spectra and Taylor and Rogers results. Noticeable differences are seen at the peaks and lower end of the spectra, which may be attributed to the different $^{\text {192}}$Ir spectra attached to the core of each source model. In addition, an energy cut of 10 keV has been applied in all GATE simulations. The $^{\text {192}}$Ir spectrum used by Taylor and Rogers was from work done by Duchemin and Coursol [30], whereas this study used the spectrum provided by the NNDC [21]. A study performed by Rivard et al. [20] showed that spectra used in both studies have minimal influence on TG43U1 parameter estimations. However, given that the NNDC spectrum is more recent and internationally evaluated, Rivard et al. suggest the NNDC $^{\text {192}}$Ir spectrum be used for all medical physics applications.

TG43U1 parameters for the GMP source have been extensively studied by both Ballester et al. [28] and Taylor and Rogers [27]. Ballester et al. conducted their study using GEANT3 whereas Taylor and Rogers used EGSnrc. Although both studies produced comparable results, Taylor and Rogers results for the radial dose function were noisy. Dose rate constants (DRC) reported in these studies were $1.118\pm 0.003$ $\text {cGy} \text {h}^{-1} \text {U}^{-1}$ [28] and $1.115\pm 0.003$ $\text {cGy} \text {h}^{-1} \text {U}^{-1}$ [27], where the average of these two values is used as $_{\text {consensus}}\Lambda$ for comparison in this work. Due to the noisy results produced by Taylor and Rogers, Ballester et al. data was used as consensus data for the remaining TG43U1 parameters ($_{\text {consensus}}\text {g}_{\text {L}}(\text {r})$ and $_{\text {consensus}}\text {F}(\text {r},\theta )$).

Perez et al. published a value of $_{\text {consensus}}\Lambda =1.117\pm 0.004$ $\text {cGy} \text {h}^{-1} \cdot \text {U}^{-1}$ for the DRC, while in this study the DRC was estimated as $\Lambda =1.123\pm 0.033$ $\text {cGy} \text {h}^{-1} \text {U}^{-1}$. The largest sources of uncertainty in this study’s best estimation of the DRC were,

- The statistical uncertainty (type A) in measuring the dose rate at P($\text {r}_{0}$, $\theta _{0}$), and
- The estimated uncertainty (type B) in interpolating between data points when estimating the mass energy absorption coefficients for all energies.

The DRC obtained in this study agrees with consensus values to within uncertainty.

Simulated radial dose function values are again in good agreement with consensus data values. All data points agreed within ±0.61%, with the largest differences being located at distances equal to 0.2 cm and 10 cm. Perez et al. [31] showed there is a relationship between phantom geometry and radial dose function and therefore a comparison was also made with Taylor and Rogers radial dose function results. Comparisons with Taylor and Rogers results agreed within $\pm 0.29\%$, with the largest difference being located at a distance equal to 6 cm. Phantom geometry used across all three studies are different, with Ballester et al. using a 40 cm high solid cylinder with a 40 cm radius [32], Taylor and Rogers using a $30 \times 30 \times 30$ $\text {cm}^{3}$ cube and a sphere with a 40 cm radius used in this study. The key result in comparing all three sets of data is that values estimated in this study are within the spread of estimations made in other studies.

Noticeable differences in anisotropy function comparisons were seen at points within 8 degrees of the source axis. Angles between 8 and 172 deg are in good agreement, with the majority of data points agreeing within 1%. Large differences near the source axis can be attributed to multiple factors. The first being a difference in source wire lengths modelled in both studies. Ballester et al. used a source wire length of 6 cm, whereas a 2 mm source wire length was used in this study. Secondly, Ballester et al. scored dose instead of kerma. By doing this, Ballester et al. were able to present more accurate results near the long axis of the source [26]. Lastly, Ballester et al. included interpolated/extrapolated data for all points within the source (including the source wire).

Overall, comparisons with consensus data are in good agreement. Dose rate constant, radial dose function and for the most part anisotropy function estimations agree within 1%. These results demonstrate that the physics, actors, VRT’s and source specifications have been defined with sufficient accuracy. Therefore GATE has shown its capability to accurately model a brachytherapy source, and the GMP source as simulated is a suitable surrogate for a real-world source.

### GATE vs ABV comparisons

As previously stated, the primary aim of this study is to assess the dose calculation accuracy of ABV in materials relevant to HDR brachytherapy using GATE V8.0 as the gold standard. Comparisons of dose as predicted by both ABV and GATE V8.0 doses were performed via Point-to-point profile comparisons and 1D gamma analysis.

Comparing point-to-point profiles, GATE and ABV doses agreed within uncertainty for the majority of data points in all profile comparisons (Fig. 2). Common trends seen in all case comparisons are the large differences seen at distances close to the source (i.e., r$\le$0.6 cm) and in low density materials such as lung and air. Large differences observed at close distances may be attributed to multiple factors, with the first being a lack of CPE. ABV and notable studies conducted by Zourari et al. [11, 13], Petrokokkinos et al. [12] and Ma et al. [14] all assume CPE exists at all points. However, CPE may not exist at points within ±0.6 cm of the source due to a nonuniform fluence [33], insufficient build up and an increase or decrease in backscatter. With high density materials such as stainless steel assigned to the source slab, differences in this region were reduced. Given the attenuation properties of materials like stainless steel, less build up material is required to reach CPE. Secondly, GATE and ABV calculated dose may differ due to voxel-size effects in high dose gradient regions. A study conducted by Taylor and Rogers [34] showed that doses may be overestimated when using a large voxel calculation size.

Large differences were also seen when estimating dose in low density materials such as air and lung. Results presented in Fig. 2a and c contradict findings in work by Zourari et al. [13] where ABV and MC lung doses were in good agreement. ABV assumes all secondary electron ranges are smaller than the voxel calculation size and deposit their energy locally [35]. Therefore in the context of cavity theory, ABV applies large cavity theory assumptions [36]. Stopping power data published by NIST [37] show that electrons with an initial kinetic energy of 0.3 MeV have a range of up to 0.842 mm in water, 3.27 mm in lung and 794 mm in air. Therefore, electron ranges will be much larger than the voxel calculation grid size and as a result $\text {K}_{\text {coll}} < \text {D}$. Energy deposited within these regions would be better described using small cavity theory, where dose is more accurately calculated using fluence and stopping powers [36]. Following these results, clinical recommendations would be to ensure that bolus, gauze and bladder filling protocols are utilised to reduce any gaps in and around the treatment area.

As a point-to-point comparison can be overly sensitive in high dose gradient regions [38] and large differences were seen in all cases at points within close proximity of the source, a gamma analysis using local normalization [38], was applied to all profiles. This collectively quantified dose differences and spatial misalignment at each data point. TG186 [4] recognises the necessity of defining gamma criteria specifically for brachytherapy and therefore propose a gamma analysis pass criteria of 2%/2 mm with a $\ge$99% pass rate. TG186 also recognises that there is limited research on gamma criteria for brachytherapy and therefore accepts that their proposed pass criteria may need adjusting depending on the case. Due to the simplicity of the phantoms used in this study, as well as the uncertainties associated with GATE’s dose estimations, a more strict gamma index criteria of 2%/1mm with a pass rate $\ge 99\%$ was deemed reasonable. Using this criteria, the results showed a significant number of failing points located in high dose gradient regions, and therefore a comparison was made with gamma passing rates using TG186’s gamma index criterion of 2%/2mm.

Of the 6 phantoms, simulations using phantoms 5 and 6 produced gamma index pass rates of 100% for both pass criteria used. However, phantoms 1, 2, 3 and 4 produced varying results. Given ABV’s inability to accurately estimate dose in low density materials, low gamma index pass rates were to be expected for phantoms 1 and 3. The key finding in the gamma analysis results is that even with high density materials producing excessive backscatter near the source, the spatial distribution of dose is within $\pm 1$ mm.

### Clinical implications

Results presented herein show that ABV can be used clinically, but with caution as ABV under-calculates dose within and beyond low density materials. This is illustrated from use of phantoms 1 and 3 (Fig. 2a, c). However, ABV demonstrates good accuracy when calculating dose in anatomical and applicator materials such as bone, cartilage, muscle, adipose, PMMA, peek, titanium and stainless steel.

Given ABV demonstrates inaccuracies in low density materials, it is recommended that brachytherapy procedures identify and minimise any air gaps immediately surrounding the source and volumes of interest. This can be achieved for gynaecological and prostate brachytherapy, for example, with Vaseline gauze and bladder filling protocols [39–41].

Despite ABV’s limitation in low density materials, it exhibits good accuracy and can be used with confidence when calculating dose in high density materials such as stainless steel. For dose calculated within and distal to stainless steel, differences between GATE and TG43U1 calculated dose ranged between −3.03 and −20.48%, and gave an average difference of −8.59%; whereas differences between GATE and ABV calculated dose ranged between −15.71 and 5.83%, and gave an average difference of 0.8%. Therefore, this study’s results showed that ABV is capable of providing accurate dose calculations for plans which include, for example, stainless-steel gynaecological applicators such as the Fletcher-suit system.

## Conclusion

This study assessed the accuracy of ABV for calculating dose to a medium with $^{\text {192}}$Ir as a brachytherapy source, as well as assessing the feasibility of using GATE in brachytherapy applications. Accuracy assessment was achieved by first constructing and validating a MC model of an $^{\text {192}}$Ir GMP source.

It was determined that result reliability would be heavily impacted by a poor simulation of the GMP source. Once the modelled source was validated, and GATE was determined to be a feasible tool, comparisons between GATE and ABV were made using point-to-point profile comparisons and 1D gamma analysis.

Data produced in this study identified two key results. The more important of these two being that ABV under-predicts dose in low-density regions. This has significant clinical implications and therefore is highly recommended to ensure that bolus, gauze and bladder filling protocols are utilised to reduce any air gaps in and around the treatment area. The second key result is that ABV accurately predicts dose in high-density regions.
